# Validation of PREdiction of DELIRium in ICu patients (PRE-DELIRIC) model for ICU delirium in general ICU and patients with liver disease: a retrospective cohort study

**DOI:** 10.1186/s40560-025-00800-3

**Published:** 2025-06-16

**Authors:** Areti Papadopoulou, Sarah L. Cowan, Jacobus Preller, Robert J. B. Goudie

**Affiliations:** 1 MRC Biostatistics Unit, School of Clinical Medicine, University of Cambridge, Cambridge, CB2 0SR UK; 2 Addenbrooke’s Hospital, Cambridge, CB2 0QQ UK; 3 Department of Medicine, University of Cambridge, Cambridge, CB2 0QQ UK

**Keywords:** Delirium, PRE-DELIRIC, Validation, Liver disease, Sedation, Opioids, Intensive care unit

## Abstract

**Background:**

Delirium, a neuropsychiatric disorder characterized by disturbances in attention, cognition, and awareness, is a common complication among intensive care unit (ICU) patients. Several predictive models have been developed that aim to identify patients at high risk of delirium. PRE-DELIRIC (PREdiction of DELIRium in ICu) and its recalibrated version, have been externally validated in several studies, but modest sample sizes have meant uncertainty remains, particularly in patient subgroups. Of particular relevance to our population (as a tertiary liver disease centre), performance in patients with liver disease has not been specifically assessed.

**Methods:**

This retrospective cohort study evaluated the PRE-DELIRIC model using data from 3312 adult ICU patients at Cambridge University Hospital, between February 2017 and September 2021. Delirium was primarily defined as either a positive Confusion Assessment Method for the ICU (CAM-ICU) or any new administration of antipsychotic medication. Predictive performance was assessed according to discrimination, measured by the area under the receiver operating characteristic (AUROC) and precision-recall curves; and calibration, as quantified by calibration slope and intercept. We also conducted subgroup analyses in patients with liver disease, sedated patients, and across varying opioid dosing.

**Results:**

Delirium occurred in 32.9% of patients. Overall, PRE-DELIRIC demonstrated moderate-to-good discriminative performance (AUROC 0.74; 95% CI 0.72–0.76); but the model significantly underpredicted delirium incidence for those patients predicted to have moderate-to-high delirium risk (PRE-DELIRIC score 0.2–0.6); and overpredicted for those predicted to be at very high risk (PRE-DELIRIC score > 0.6). Among patients with liver disease (41.6% delirium incidence), discrimination was similar to the overall cohort (AUROC 0.73; 95% CI 0.66–0.81), but calibration was poor, with significant under-prediction of delirium. Discrimination was significantly poorer in both sedated patients and patients receiving high opioid dosing.

**Conclusion:**

This is the largest validation study of the PRE-DELIRIC model to date, and the first to specifically consider patients with liver disease. We found moderate-to-good discriminative predictive performance both overall and in liver disease patients, but calibration was only moderate overall, and significantly under-predicted risk in patients with liver disease. Recalibration of the model and further subgroup-specific adjustments may enhance its utility in clinical practice.

**Supplementary Information:**

The online version contains supplementary material available at 10.1186/s40560-025-00800-3.

## Introduction

Delirium is a neuropsychiatric disorder characterised by disruption in attention, cognition and awareness [1, 2]. It is a common complication of intensive care unit (ICU) admission, with reported incidence between 20 and 58% [3–8], and is known to be associated with adverse outcomes, including prolonged length of stay, higher rates of subsequent cognitive impairment, and mortality [1, 9].

Delirium can be distressing for patients and their families, and places an increased burden on ICU services due to the increased length of stay [10]. ICU guidelines therefore focus on strategies thought to reduce the risk of delirium [10]. These include early mobilisation, analgesia, daily interruption of sedation or limiting the depth of sedation and sleep promotion. While some interventions should be routinely employed for all patients, others may involve limited resources (e.g. allocation of individual rooms with natural light), be time or labour intensive (frequent reorientation and early mobilisation) or in the case of pharmacological prevention may expose the patient to side effects from the drugs used. These strategies are therefore best targeted at patients at highest risk of delirium.

To date, three models for predicting delirium in ICU patients have been the focus of clinical research [11]. PRE-DELIRIC [7] and Lanzhou [12] employ data gathered 24 h after ICU admission, whereas E-PRE-DELIRIC (Early PREdiction of DELIRium in ICu patients) [13] uses only information at the point of admission. PRE-DELIRIC was developed in the Netherlands [7], subsequently recalibrated in a multinational study [8] and has been externally validated in a number of studies [3–6, 14–16].

Despite the validation studies of the PRE-DELIRIC model in many countries [3–6, 14–16], considerable uncertainty about its performance remains. Reported area under the receiver operating characteristic (AUROC) ranges widely from 0.70 to 0.84 [3–6, 14–16], reflecting a wide range of discriminative performance. This may in part be due to the modest sample sizes employed: between 38 and 2724 patients in the respective ICUs [3–6, 14–16]. The modest sample sizes of these studies have also limited the ability of previous studies to assess performance in clinical subgroups.

The study ICU supports a large secondary and tertiary liver disease service and liver transplant programme. Previous studies have not specifically considered patients with liver disease, who form an important part of our cohort, and we therefore sought to validate its performance in this group of patients. Consensus guidelines describe acute encephalopathy (which may include hepatic encephalopathy) as a rapidly developing pathological brain process which may manifest in the clinical features of delirium [17]. One can postulate that, in addition to the pathophysiological processes usually associated with delirium [18], patients with liver disease also have the risks associated with liver dysfunction which include the role of hyperammonemia. Ammonia can cross the blood–brain barrier, decrease excitatory neurotransmission and together with glutamate be converted by astrocytic into glutamine, which as an osmolyte can cause brain oedema [19]. Patients with liver disease may therefore have additional risk factors for delirium, as compared with general ICU patients, but these are not comprehensively represented in the PRE-DELIRIC scoring algorithm. The performance of PRE-DELIRIC may therefore differ in this group of patients, but this has not previously been assessed in any validation of PRE-DELIRIC.

In this study, we conducted the largest validation study of PRE-DELIRIC to date in patients admitted to a UK general ICU. The large sample size of our study allows us to assess performance in clinical subgroups that have not been previously assessed. Specifically, we evaluated the performance of PRE-DELIRIC in patients with underlying liver disease, sedated patients and those receiving varying doses of opioid medications.

## Methods

We report our findings according to the Transparent Reporting of a multivariable prediction model for Individual Prognosis or Diagnosis reporting guidelines (TRIPOD) statement [20].

### Study design

We performed a retrospective cohort study using the electronic medical records of all patients admitted to the general ICU at Addenbrooke’s hospital, Cambridge University Hospitals (CUH), between 1 February 2017 and 30 September 2021. CUH is a large teaching hospital, providing emergency and elective, medical and surgical care to a large diverse population [21]. CUH is also a leading liver transplant centre and provides tertiary hepatology services for the East of England.

### Study population

All adults (≥ 18 years of age) admitted to the ICU during the study period were eligible for inclusion. The following exclusion criteria were applied according to the original study: (1) patients who developed delirium within the first 24 h of ICU admission, (2) ICU admission less than 24 h, and (3) patients who were persistently comatose (having Richmond Agitation-Sedation Scale (RASS) score of − 4 or − 5) throughout the first 24 h in the ICU [22]. We additionally exclude: (4) readmissions, as recorded in Intensive Care National Audit & Research Centre (ICNARC) audit data [23].

### Delirium assessment

ICU patients were assessed for delirium twice daily by critical care nurses as part of routine clinical care, using the Confusion Assessment Method for the Intensive Care Unit (CAM-ICU) assessment [24]. CAM-ICU is a standardised evidence-based tool for delirium assessment in the ICU [25]. Delirium was defined in the primary analysis as either a positive CAM-ICU assessment or the new administration of any antipsychotic medication during the patient’s ICU stay. While the original studies included only haloperidol in the definition of delirium [7, 8], we also included atypical antipsychotic drugs since both have been previously recommended by our ICU guidelines for the management of agitated delirium [6]. We also consider alternative definitions of delirium in sensitivity analyses (see Statistical analysis section below). RASS was recorded hourly in the ICU.

### Predictors

Clinical data were extracted from Epic, the integrated electronic health record system used at the study hospital [26], and linked ICNARC audit records. All data were recorded in the electronic health records system by physicians and nurses in the participating ICUs, and were extracted by a clinical informatics team who did not participate in clinical care.

We aimed to reproduce the definitions from the original study as closely as possible. PRE-DELIRIC is comprised of ten predictors, measured in the first 24 h of an ICU admission: age, Acute Physiology and Chronic Health Evaluation II (APACHE II) score, coma category, admission category, infection, metabolic acidosis, morphine use, sedative use, urgent admission and serum urea [7]. Age, admission APACHE II score, admission category and urgency of admission were extracted from local data collected for ICNARC [23]. Medication usage was extracted from the electronic medication administration record in Epic [26]. Infection status was defined as any use of any antibacterial medication during the first 24 h of the ICU stay, reflecting clinician belief on whether infection was present. In the study ICU the use of antibiotics is discussed in a daily multi-disciplinary meeting with a Microbiologist. Sedation status was defined as any administration of propofol, midazolam or lorazepam. A patient was diagnosed with metabolic acidosis if the pH was under 7.35 and base excess was under −2. The highest serum urea measurement during the first 24 h in the ICU stay was used.

Comatose patients (defined as RASS of −4 or −5) who were administered any of fentanyl, remifentanil, propofol or midazolam were eligible for classification as “comatose with use of medication”. Non-pharmacological causes of coma were identified from ICNARC data. Comatose patients with both pharmacological and non-pharmacological causes for coma were classified in the “combination” category. While the original PRE-DELIRIC model specifies “morphine usage” as a predictor [7], the most commonly used opioids in the study ICU are fentanyl and remifentanil, so we converted opioid administration into morphine equivalent doses [27].

### Sample size

All available data in the database, during the study period, were used to maximise the power and generalisability of the results [28].

### Missing data

Only two risk factors had missing values; metabolic acidosis and urea. Metabolic acidosis had 17 (0.5%) missing values that were imputed as no acidosis present. Urea had 11 (0.3%) missing values and were imputed with the mean value of the respective group; delirious or not delirious.

### Statistical analysis

For all analyses presented in the current study, we used R statistics version 4.1.3 [29]. For the clinical characteristics of ICU patients, mean (SD) was employed for normally distributed characteristics, whereas the median (interquartile range (IQR)) was used for non-normally distributed characteristics. Binary and multiple categorical risk factors are presented as frequency and percentage.

In the primary analyses, we used the latest version of the PRE-DELIRIC model, specifically the “recalibrated coefficients” version [8]. The predictive ability of the PRE-DELIRIC model was evaluated based on both discrimination and calibration. Regarding the discrimination ability, the AUROC curve was employed. We also plot the precision-recall (PR) curve, showing precision (positive predictive value (PPV)) against recall (sensitivity), and calculate the area under the PR curve (AUPRC). Calibration was assessed by examining the agreement between predicted and observed values, and the calibration intercept and slope from the model logit(probability of delirium) = alpha + beta × logit(*p*), where *p* is the PRE-DELIRIC score [20].

As sensitivity analyses, we examined performance when delirium was defined as either a positive CAM-ICU assessment or administration of haloperidol only (i.e. not including other antipsychotic medications); and when delirium was solely defined by CAM-ICU assessments (not including administration of any antipsychotic medications). We also examined performance using the original model coefficients [7], rather than the recalibrated coefficients; and assessed a strict definition of “morphine use” that included only administration of morphine itself (i.e. we exclude other opioids) to exactly match the original PRE-DELIRIC paper [7].

As secondary analyses, we assessed PRE-DELIRIC performance in three specific subgroups of ICU patients in CUH: (1) patients with liver disease who had not had a liver transplant; (2) patients who received sedative medication and (3) patients who received varying doses of opioid drugs.

We also explored relationships between key clinical variables and delirium incidence, including ammonia levels, bilirubin concentration and opioid administration.

### Ethics approval

The study was approved by a UK Health Research Authority ethics committee (19/SC/0541). Patient consent was waived because the de-identified data presented here were collected during routine clinical practice; there was no requirement for informed consent.

## Results

### General characteristics

**Fig. 1:**
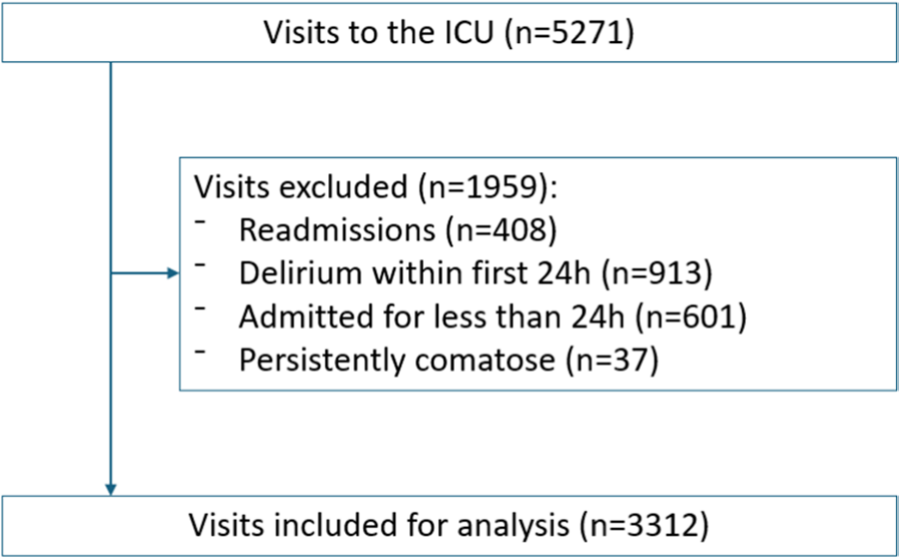
Flowchart of exclusions

In total, there were 5271 ICU visits during the study period, with 1959 excluded according to the exclusion criteria (Fig. 1).

Patient characteristics for the remaining 3,312 ICU visits included in our study are presented in Table 1. The mean age of patients was 59 years and 1,959 (59.1%) were male. The mean admission APACHE II score was 16. The majority of the patients were admitted for medical reasons (57.4%) and the vast majority were urgent admissions (85.1%). Most patients were administered antibiotics (68.9%) and opioids (66.2%) during the first 24 h in the ICU. Median length of ICU stay was 3.9 days, with 11.6% (383) ICU mortality.

**Table 1:** Clinical characteristics of ICU patients in CUH

Characteristics	
Age (years), mean (SD)	59 (17.1)
Male, *n* (%)	1,959 (59.1)
APACHE-II score at admission, mean (SD)	16 (5.8)
Admission category, *n* (%)	
Surgical	1,044 (31.5)
Medical	1,901 (57.4)
Trauma	70 (2.1)
Neurological	297 (9.0)
Mechanical ventilation, *n* (%)	1,753 (52.9)
ICU length of stay (days), median (IQR)	3.9 (2.1–7.9)
ICU mortality, *n* (%)	383 (11.6)
Delirium, *n* (%)	1,091 (32.9)

Whilst on ICU (after excluding those who developed delirium during the first 24 h), 1,091 (32.9%) of patients developed delirium according to our definition; 1,041 were identified from the CAM-ICU assessments and 50 were identified from the administration of an antipsychotic medication. The complete set of PRE-DELIRIC predictors are presented in Supplementary Table 1. More than half (52.7%) of patients in our cohort received opioids equivalent to more than 18.7 mg of morphine, placing them in the highest category for this predictor in PRE-DELIRIC.

### Primary analysis

**Fig. 2:**
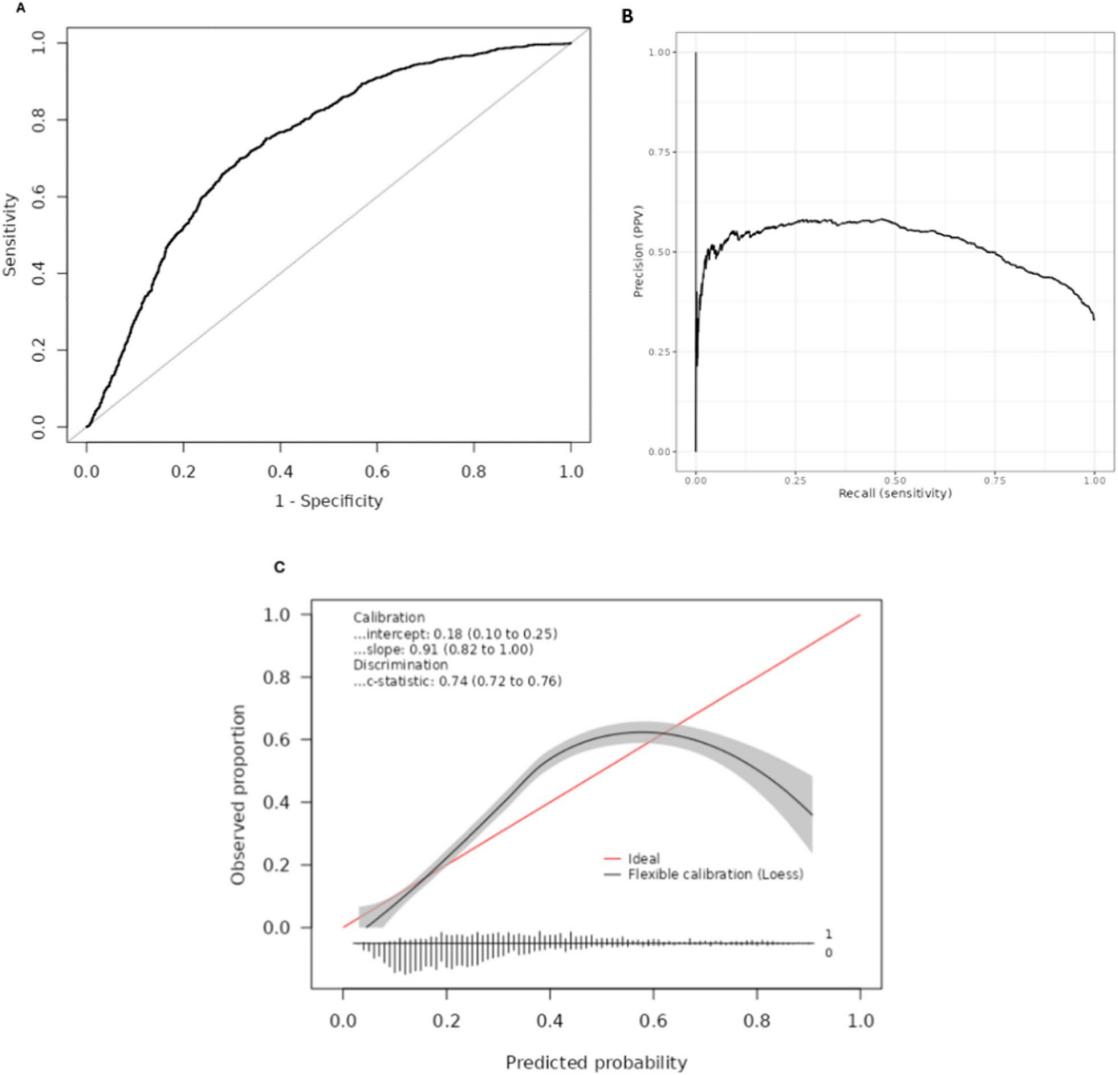
**A** Receiver operating characteristics (ROC) of PRE-DELIRIC model (area under the ROC 0.74; 95% confidence interval 0.73–0.76). **B** Precision-Recall (PR) curve of PRE-DELIRIC model (area under the PR curve 0.52). **C** Calibration plot for the PRE-DELIRIC model. At the bottom of the plot, histogram of the predicted risks are shown for ptients with (1) delirium and without (0) delirium. All three plots correspond to all included patients

The AUROC was 0.74 (95% CI 0.72–0.76), which can be considered a moderate-to-good performance [30] (Table 2) (Fig. 2A). The histogram of PRE-DELIRIC scores, shows that for patients with predicted risk above 50%, there is an extensive overlap for those who did and did not develop delirium, indicating that PRE-DELIRIC is not able to fully discriminate patients (Supplementary Fig. 1). The AUPRC was 0.52 (Fig. 2B) indicating the average PPV across all sensitivity values was notably higher than delirium incidence (32.9%). In particular, for sensitivity between 0.03 and 0.74, the PPV was above 0.50, indicating delirium occurred in at least half of the patients that were predicted to develop delirium in this range.

**Table 2:** Main and sensitivity analyses using the PRE-DELIRIC model in CUH’s data

	Sample size	Delirium incidence	AUROC (95% CI)	AUPRC
Primary analysis	3312	1,091 (32.9%)	0.74 (0.72–0.76)	0.52
Sensitivity analyses				
Original PRE-DELIRIC [7]	3312	1,091 (32.9%)	0.74 (0.72–0.76)	0.52
Delirium defined by CAM-ICU or haloperidol administration	3312	1,068 (32.2%)	0.74 (0.73–0.76)	0.51
Delirium defined only from CAM-ICU	3312	1,041 (31.4%)	0.75 (0.73–0.76)	0.51
Strict “morphine use” definition (as per van den Boogaard et al. [7])	3312	1,091 (32.9%)	0.72 (0.71–0.74)	0.50
Subgroup analyses				
Liver disease patients	173	72 (41.6%)	0.73 (0.66–0.81)	0.58
Sedated patients	1775	891 (50.2%)	0.60 (0.57–0.62)	0.55
*Morphine equivalent use*				
No opioids	1120	138 (12.3%)	0.65 (0.60–0.69)	0.19
Top decile (1469 mg to 4363 mg opioids)	219	139 (63.5%)	0.58 (0.50–0.66)	0.70

AUROC: Area Under the Receiver Operating Characteristic curve

CI: Confidence Interval

AUPRC: Area Under the Precision–Recall Curve

Figure 2C shows that the model significantly underpredicted delirium incidence for those patients predicted to have moderate-to-high delirium risk (PRE-DELIRIC score 0.2–0.6); and overpredicted for those predicted to be at very high risk (PRE-DELIRIC score > 0.6). The calibration slope was beta 0.91 (95% CI 0.82–1.00), indicating that PRE-DELIRIC overestimates the variability in delirium incidence. In other words, the actual incidence of delirium in patients with low and high PRE-DELIRIC scores is more similar than would be the case in an ideal (well-calibrated) prediction model. Furthermore, the calibration intercept was alpha 0.18 (95% CI 0.10–0.25), suggesting that the model tends to overestimate delirium incidence at low PRE-DELIRIC score levels (Fig. 2C).

### Sensitivity analyses

The discrimination of the original PRE-DELIRIC model [7] was identical to the recalibrated PRE-DELIRIC model [8] that we used in our primary analysis, as was the AUPRC (Table 2). As might be expected, calibration in the original model was worse than the recalibrated model (calibration slope beta 0.42; calibration intercept alpha − 1.30; Supplementary Fig. 2).

Modifications in the definition of delirium led to little change in performance. Defining delirium as either a positive CAM-ICU or the administration of haloperidol gave delirium incidence 32.2%, with AUROC 0.74 (95% CI 0.73–0.76) (Table 2) (Supplementary Fig. 3), while defining delirium solely from CAM-ICU assessments gave delirium incidence 31.4%, with AUROC 0.75 (95% CI 0.73–0.76) (Table 2). Both gave similar calibration (Supplementary Figs. 3, 4).

Using the strict morphine use definition dramatically increased the proportion of patients classified as receiving no morphine (from 33.8 to 85.4%) and decreased the proportion receiving the highest category (52.7% to 0.8%) (Supplementary Table 2). Using this definition the AUROC was 0.72 (95% CI 0.71–0.74) (Table 2), non-significantly lower than the primary analysis. Calibration was similar to the overall calibration (Supplementary Fig. 5).

### Subgroup analyses

We evaluated the PRE-DELIRIC model in several subgroups of ICU patients.

#### Liver disease patients

**Fig. 3:**
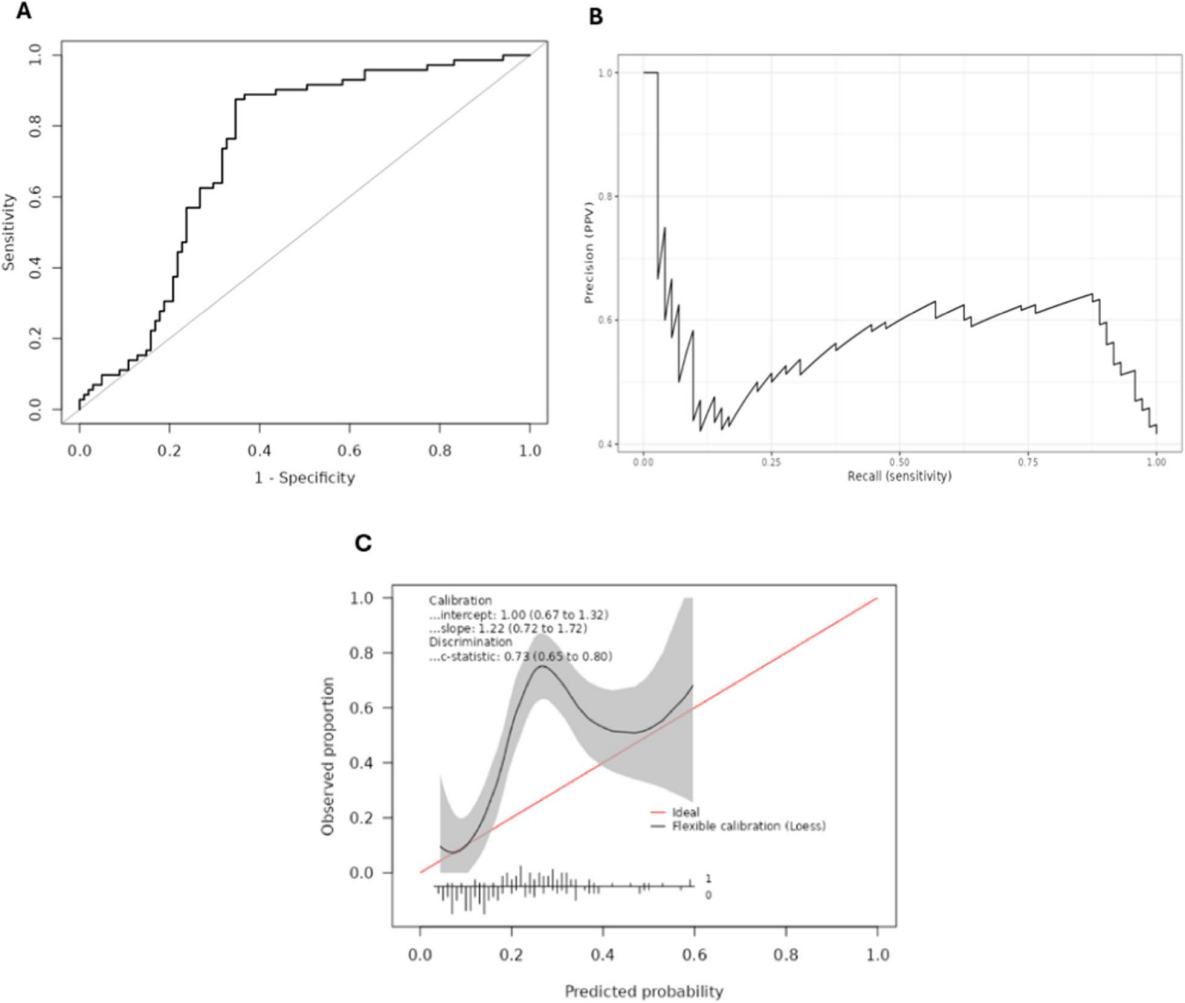
Performance characteristics in the subgroup of patients with liver disease who had not received a liver transplant. **A** Receiver operating characteristics (ROC) of PRE-DELIRIC model (area under the ROC 0.73; 95% confidence interval 0.66–0.81). **B** Precision-Recall (PR) curve of PRE-DELIRIC model (area under the PR curve 0.58). **C** Calibration plot for the PRE-DELIRIC model. At the bottom of the plot, histogram of the predicted risks are shown for patients with (1) delirium and without (0) delirium

There were 173 patients admitted with liver disease who had not received a liver transplant. Incidence of delirium in this group was 41.6%, considerably higher than in the overall cohort. Overall discriminative performance in this group was similar to the whole cohort (AUROC 0.73; 95% CI 0.66–0.81) (Table 2) (Fig. 3A). However, calibration was poor in patients with PRE-DELIRIC score above 0.18; delirium was strongly underpredicted in this group (Fig. 3C).

None of the PRE-DELIRIC predictors capture liver dysfunction, so we conducted an exploratory analysis to assess whether bilirubin or ammonia might assist prediction of delirium. As shown in Supplementary Figs. 6 and 7, this suggested that for both bilirubin and ammonia, higher levels may be associated with higher delirium incidence.

#### Sedated patients

**Fig. 4:**
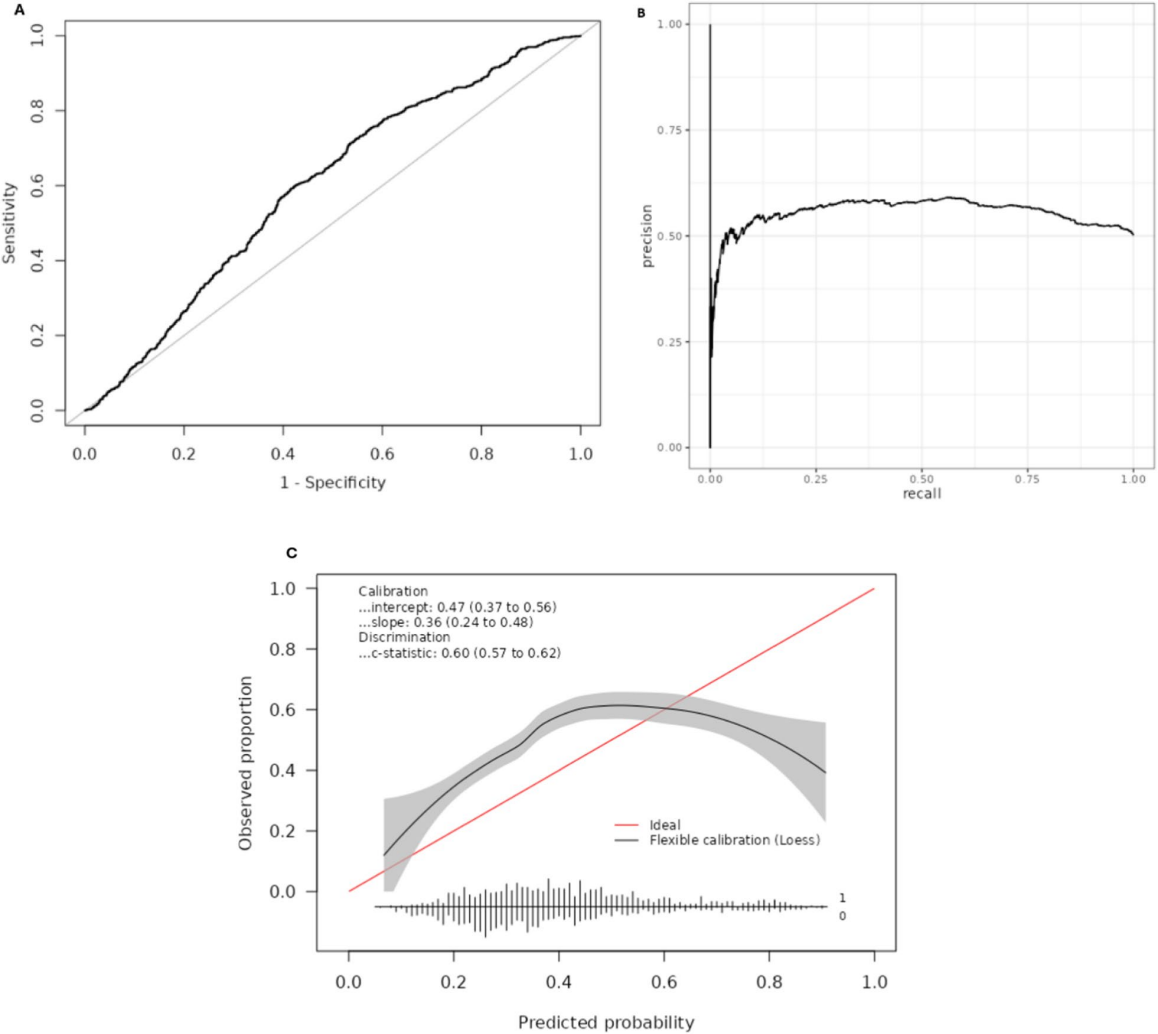
Performance characteristics in the subgroup of patients who received sedative drugs. **A** Receiver operating characteristics (ROC) of PRE-DELIRIC model (area under the ROC 0.60; 95% confidence interval 0.57–0.62). **B** Precision-Recall (PR) curve of PRE-DELIRIC model (area under the PR curve 0.55). **C** Calibration plot for the PRE-DELIRIC model. At the bottom of the plot, histogram of the predicted risks are shown for patients with (1) delirium and without (0) delirium

Incidence of delirium in the 1775 patients who received sedative drugs was 50.2%, considerably higher than the wider study cohort. Discriminative performance was significantly lower than the wider study cohort, with AUROC 0.60 (95% CI 0.57–0.62) (Table 2) (Fig. 4A). The calibration plot showed that PRE-DELIRIC significantly underpredicted delirium incidence for those with PRE-DELIRIC score between 0.12 and 0.60 (Fig. 4C).

#### Opioid use

Delirium incidence was strongly associated with administration of opioids, with increasing dosage associated with increasing incidence of delirium: increasing from 16.4% within the lowest decile (0.2 mg/day to 3.3 mg/day morphine equivalent) to 63.5% in the highest decile (1470 mg to 4363 mg morphine equivalent) (Supplementary Fig. 8). Detailed delirium incidence per decile of morphine, fentanyl and remifentanil equivalent dosing for ICU patients in CUH can be found in Supplementary Fig. 9.

Additionally, we examined the performance of the PRE-DELIRIC model per decile of morphine equivalent drugs. Detailed results, including AUROC and AUPRC per morphine equivalent dosing decile, can be found in Supplementary Table 3 (Supplementary Fig. 10). The calibration of the model is strongly affected by use of opioids. PRE-DELIRIC significantly overpredicts delirium in patients with no opioid use (calibration intercept alpha −0.44 [95% CI −0.63, −0.26] (Supplementary Fig. 11)), and significantly underpredicts delirium at the highest morphine decile (calibration intercept alpha 1.11 [95% CI 0.82, 1.40] (Supplementary Fig. 12)). Discriminative performance was significantly lower in these subgroups than in the overall cohort (Table 2).

## Discussion

This study provides the largest validation study to date of the PRE-DELIRIC risk prediction score. We found that in our cohort, PRE-DELIRIC showed moderate-to-good discrimination with an AUROC of 0.74. This is similar to what has been reported in previous validation studies [3–6, 14–16]. The PPV of PRE-DELIRIC was above 0.5 at most sensitivities which, in the context of 32.9% incidence, suggests that overall PRE-DELIRIC provides some information to inform clinical decision making. However, we found that for the majority of our patients, PRE-DELIRIC under-predicted the risk of delirium, indicating that calibration could be improved.

While the performance in the overall cohort was acceptable, this masks significant variation between the performance in different groups of patients.

This study represents the first to look specifically at the performance of the model in patients with liver disease. In this analysis, we included patients who were admitted to the ICU because of liver pathology, excluding those who had undergone an elective liver transplant (patients would typically be stabilised before progressing to a liver transplant and have less exposure to acute liver dysfunction in context of good graft function, which would be expected in the majority of liver transplant recipients). We found that in these patients PRE-DELIRIC showed very good discrimination, with a sharp rise in incidence of delirium associated with a rise in PRE-DELIRIC score. However, the model was significantly under-calibrated with a very significantly increased risk of delirium observed in our patients, suggesting that clinical utility for patients with liver disease would be improved by recalibration of PRE-DELIRIC.

Given that liver dysfunction is not captured anywhere within PRE-DELIRIC, we undertook some exploratory analysis to assess whether bilirubin or ammonia might assist prediction of delirium. However, for both tests, the number of patients at very high levels was small and therefore confidence intervals for the rate of delirium are wide. In addition, as ammonia is not routinely measured and will often be sent because hepatic encephalopathy is suspected, this will be subject to confounding from missing data and needs further analysis.

Among sedated patients, in whom the incidence of delirium was much higher (50.2%), we have shown that the model performs considerably more poorly. In this group, AUROC was only 0.60 (95% CI 0.57–0.62) and the model considerably under-predicted the risk of delirium for the majority of patients. This may be because sedation is in itself an important risk factor for the development of delirium (indeed the incidence of delirium was 50.2% in patients who received sedation within the first 24 h, compared to 32.9% in the overall cohort). In this population, regardless of the PRE-DELIRIC cutoff chosen, the positive predictive value remains around 55%, which is only slightly higher than the incidence of delirium in this population (50.2%). This means that the PRE-DELIRIC score adds very little information about the likelihood of a sedated patient developing delirium, and therefore, clinical utility of the model in this group is highly limited.

Another group in whom the model under-performed were the patients who were administered high doses of opioids. The original study included the amount of morphine administered as one of the predictors associated with an increased risk of delirium. In our unit, we use relatively little morphine, with the most commonly used opioids over the study time-period being fentanyl and remifentanil. Despite the different pharmacokinetics of these synthetic opioids when compared to morphine, we found that the risk of delirium increased as the dose of fentanyl or remifentanil increased, and indeed that this relationship continued at vastly higher morphine-equivalent doses than described in the original study. This is illustrated by the progressive rise in the rate of delirium according to deciles of morphine-equivalent dosing, along with the overprediction of the rate of delirium at low morphine-equivalent doses, and underprediction at the highest doses (Supplementary Figs. 8, 11, 12). Given this finding, we believe that calibration of the model could be improved by refitting to allow higher categories of morphine-equivalent dosing, such as commonly found when using synthetic opioid infusions.

Our study has several strengths. As the largest validation of PRE-DELIRIC to date, we were able to provide smaller uncertainty bounds than previous studies, and to analyse performance in several subgroups. In particular, we were able to consider patients with liver disease, who have not been specifically assessed previously. A further strength is that we had remarkably little missing data within the predictors included and so were able to accurately calculate the PRE-DELIRIC score for the vast majority of patients.

Our study also has several weaknesses. A major weakness of this study is that it is from a single hospital, making generalisability beyond this setting less clear. In addition, compared to the original PRE-DELIRIC paper, we have comparatively few neurology and trauma patients due to the existence of a dedicated neuro-critical care unit within the same hospital. As with any retrospective study based on routinely collected data, the data used in our analysis rely on the quality of the data that was entered at the time, however given that they reflect the documentation of CAM-ICU on our unit, this can be considered to reflect the performance of PRE-DELIRIC in a real-world setting. Finally, we used administration of antibiotics as a proxy for infection. In surgical patients, this may classify patients receiving prophylactic antibiotics as infected. As a sensitivity analysis, we classified surgical patients as infected only if they continued to receive antibiotics between 24 and 48 h after ICU admission: this made no difference in the performance of PRE-DELIRIC (results not shown).

## Conclusions

Our study represents the largest validation of the PRE-DELIRIC model to date in a UK general ICU, demonstrating its utility as a moderate-to-good predictor of delirium. While the model performs well overall, subgroup analyses in patients with liver disease, sedated patients, and those receiving opioid medications indicate reduced discrimination and poor calibration, limiting clinical utility in these subgroups. Inclusion of markers of liver dysfunction and inclusion of higher opioid dose categories may improve the performance of the PRE-DELIRIC model.

## Supplementary Information

**Additional file 1.**

**Additional file 2.**

## Data Availability

The data that support the findings of this study are available from Cambridge Clinical Informatics, but restrictions apply to the availability of these data, which were used under license for the current study, and so are not publicly available. The data are anonymised, but to preserve patient confidentiality and privacy, the Data Use Agreement states that the data cannot be deposited into open access repositories of any kind. Anyone wishing to access data must submit and receive approval for access to these data from the Cambridge Clinical Informatics Research Data Governance Committee.

## Abbreviations

APACHE II: Acute Physiology and Chronic Health Evaluation II
AUROC: Area under the ROC curve
AUPRC: Area under the PR curve
CAM-ICU: Confusion Assessment Method for the ICU
CUH: Cambridge University Hospitals
E-PRE-DELIRIC: Early PREdiction of DELIRium in ICu patients
ICNARC: Intensive Care National Audit & Research Centre
ICU: Intensive care unit
IQR: Interquartile range
PPV: Positive predictive value
PR: Precision-recall
PRE-DELIRIC: Early PREdiction of DELIRium in ICu patients
RASS: Richmond Agitation-Sedation Scale
ROC: Receiver operator characteristic
SD: Standard deviation
TRIPOD: Transparent Reporting of a multivariable prediction model for Individual Prognosis or Diagnosis reporting guidelines
