# Supplementary material for: Validation of PREdiction of DELIRium in ICu patients (PRE-DELIRIC) model for ICU delirium in general ICU and patients with liver disease: a retrospective cohort study
Source: J Intensive Care. 2025 Jun 16;13:33. doi: 10.1186/s40560-025-00800-3 (PMC12168250; doi:10.1186/s40560-025-00800-3)
Supplement: Supplementary file 1 — Additional file 1. [file 40560_2025_800_MOESM1_ESM.docx]

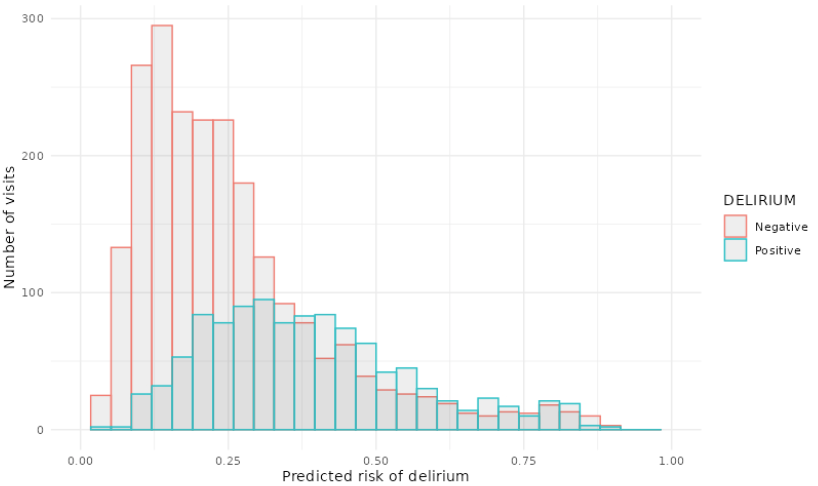


Supplementary Figure 1: Histogram of predicted risk of delirium by delirium status.


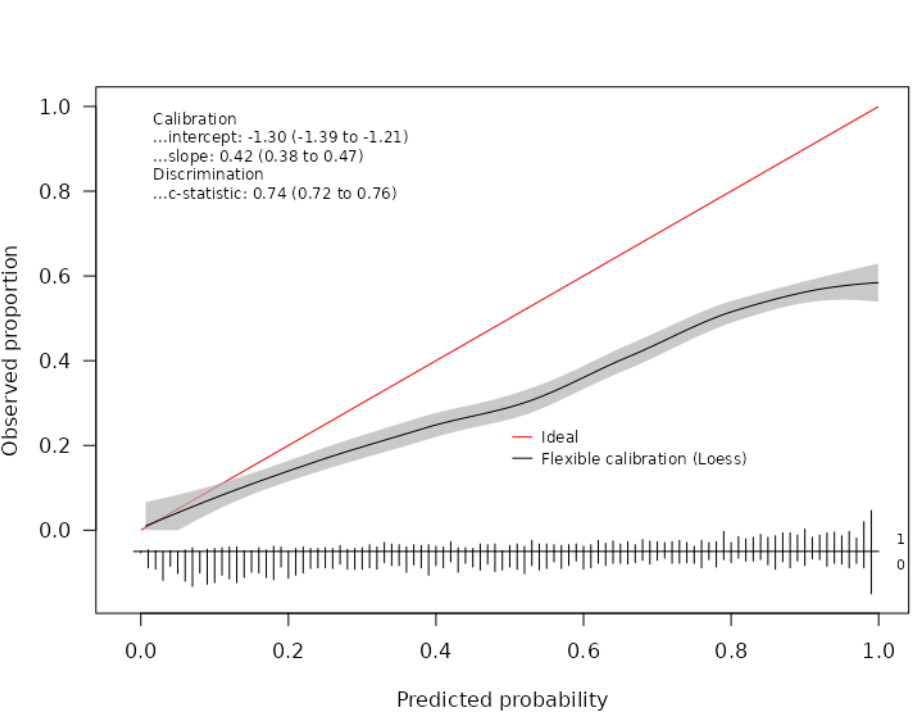


Supplementary Figure 2: Calibration plot for the original PRE-DELIRIC model [1] (Table 2).


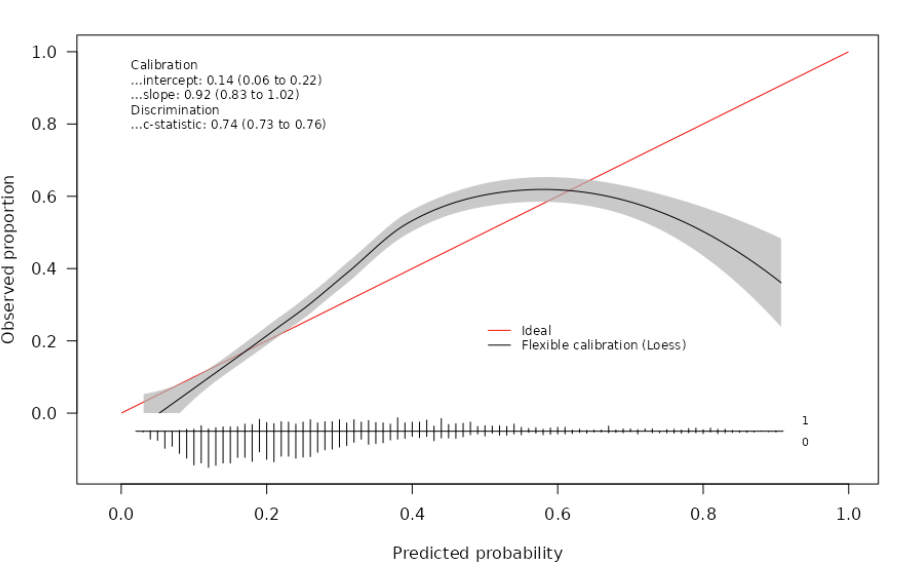


Supplementary Figure 3: Calibration plot for the PRE-DELIRIC model when delirium was defined from CAM-ICU assessments or haloperidol administration (Table 2).


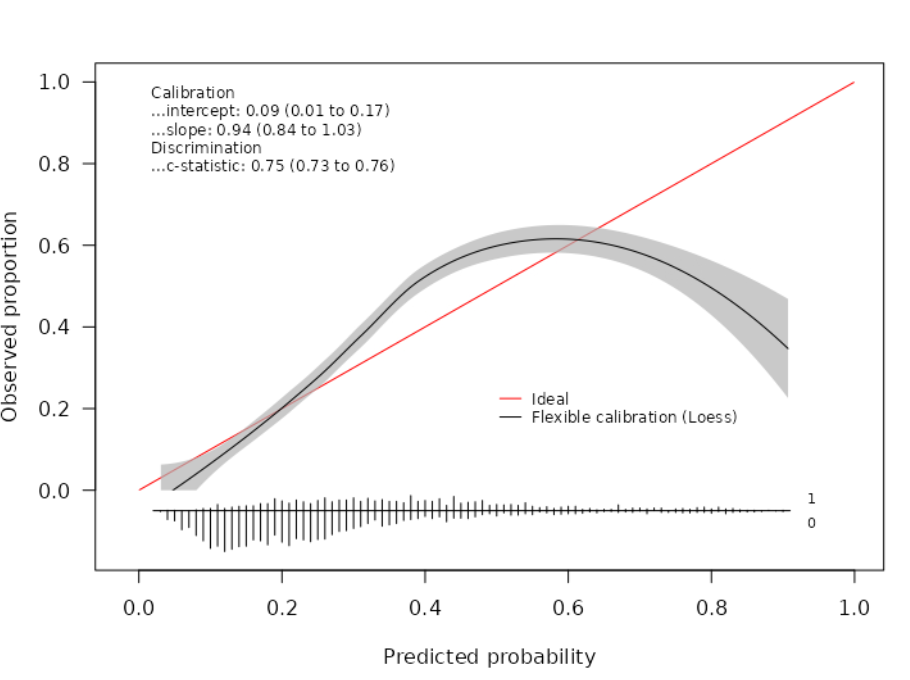


Supplementary Figure 4: Calibration plot for the PRE-DELIRIC model when delirium was defined only from CAM-ICU assessments (Table 2).


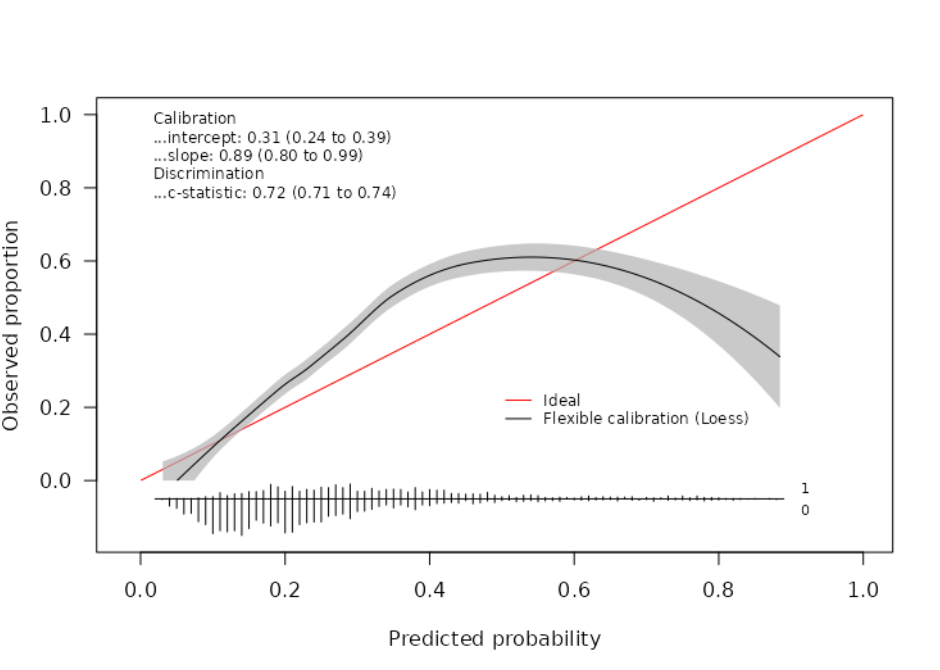


Supplementary Figure 5: Calibration plot for the PRE-DELIRIC model by extracting the strict morphine use definition as in the original paper [1] (Table 2).


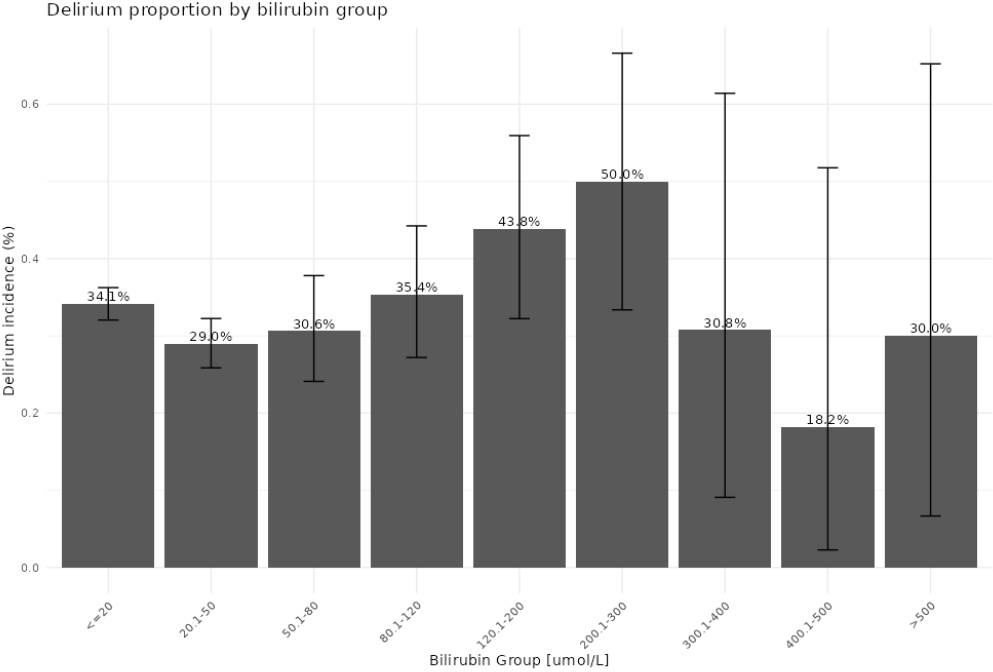


Supplementary Figure 6: Prevalence of delirium for different bilirubin groups, with binomial C.I., for ICU patients in CUH.


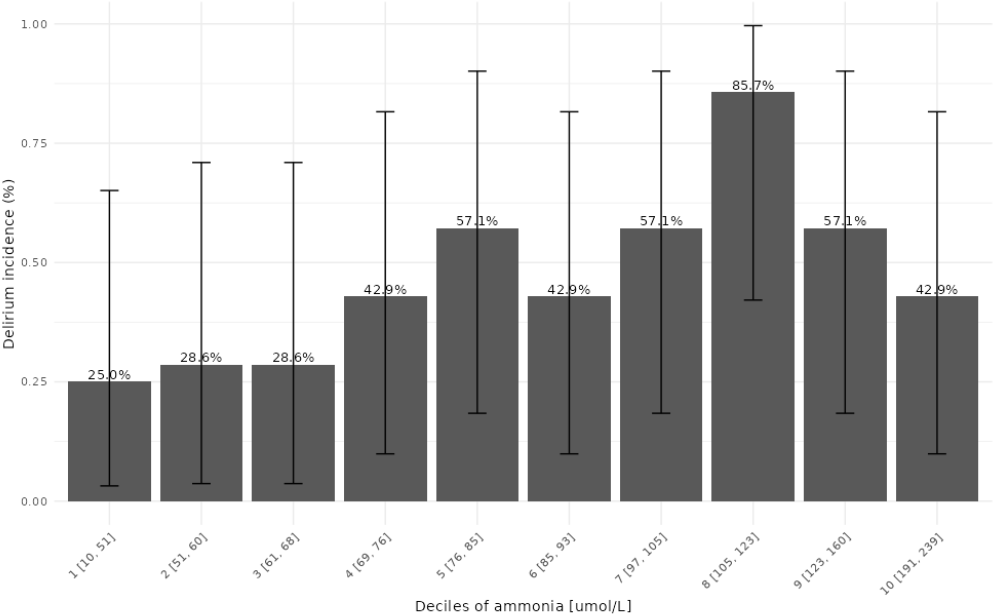


Supplementary Figure 7: Prevalence of delirium per deciles of ammonia, with binomial C.I., for ICU patients in CUH.


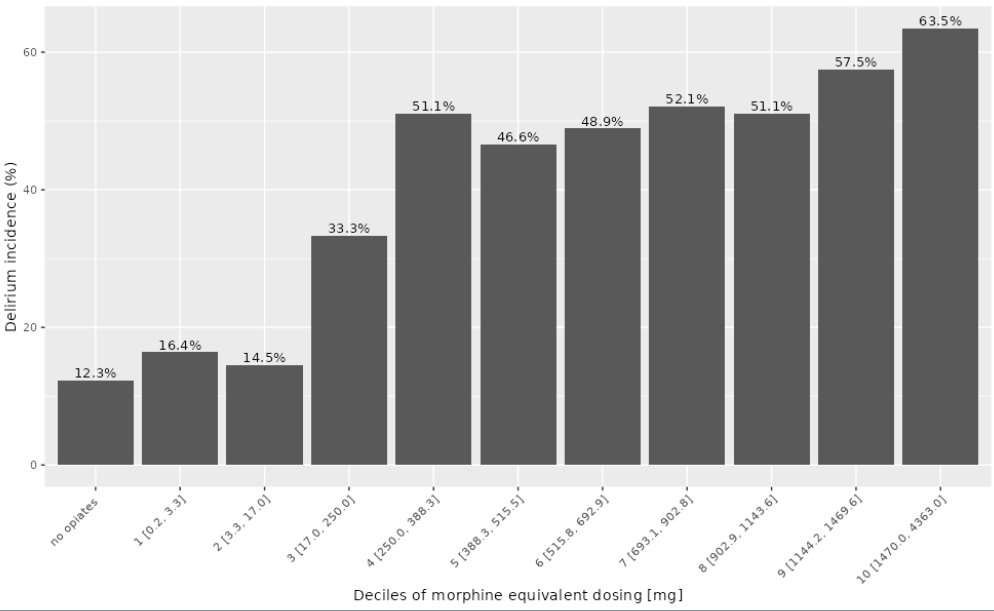


Supplementary Figure 8: Incidence of delirium per decile of morphine equivalent dosing (total morphine, fentanyl, remifentanil) for ICU patient’s in CUH.


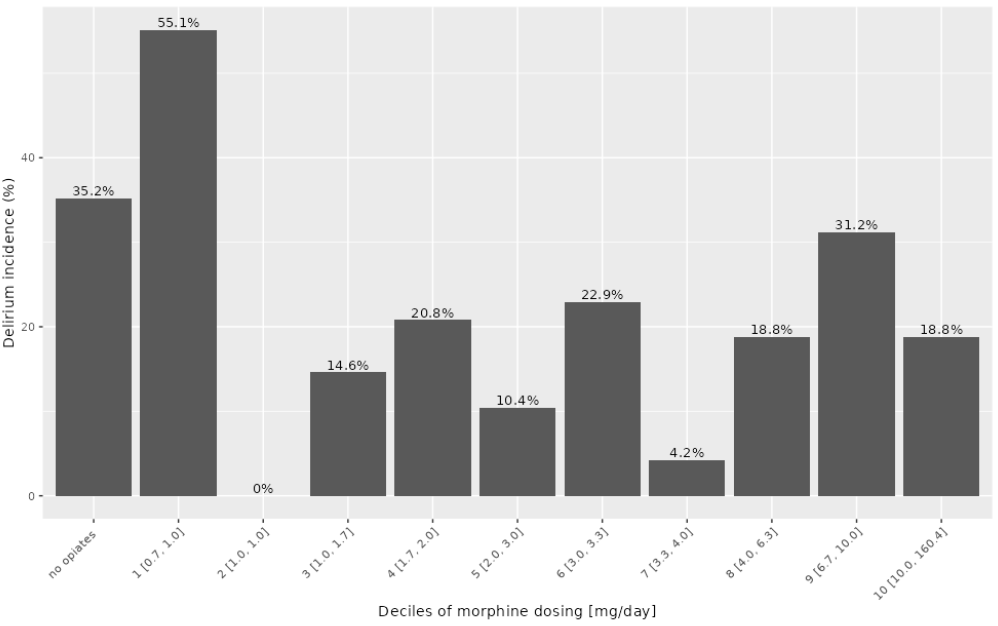


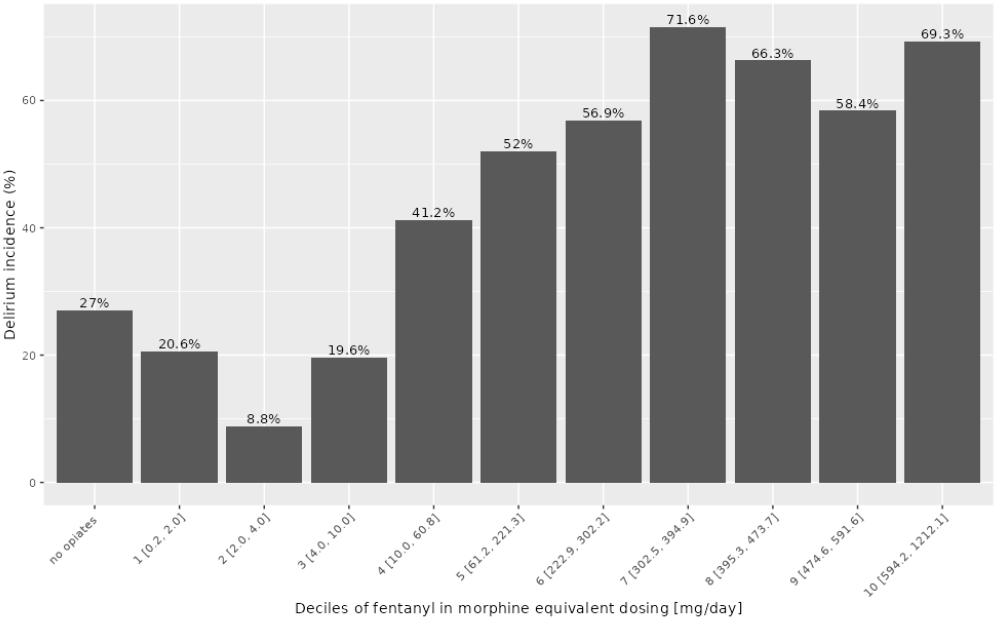


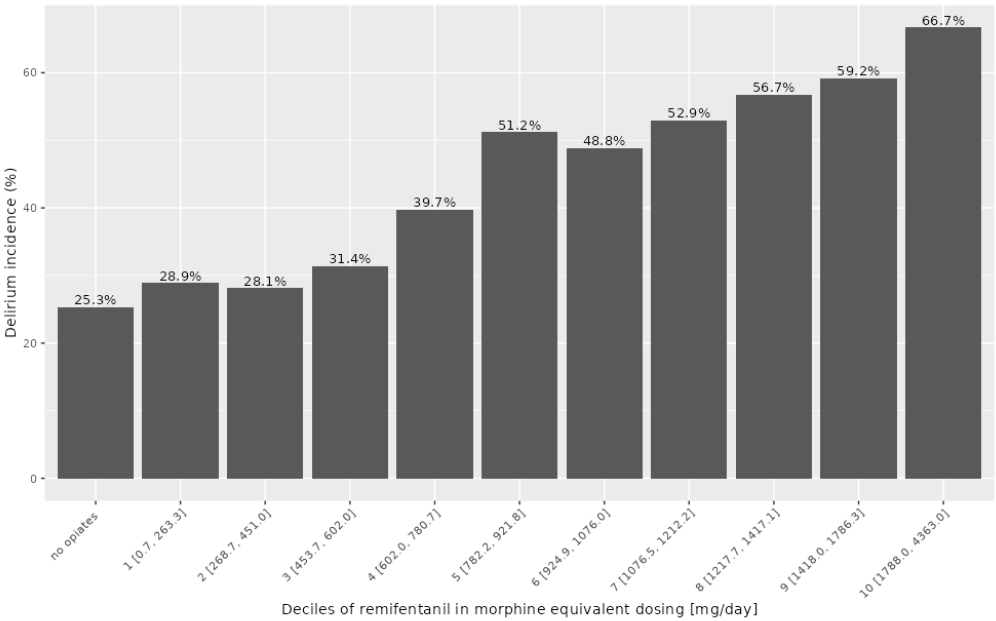


Supplementary Figure 9: Incidence of delirium per decile of morphine, fentanyl and remifentanil equivalent dosing for ICU patient’s in CUH.


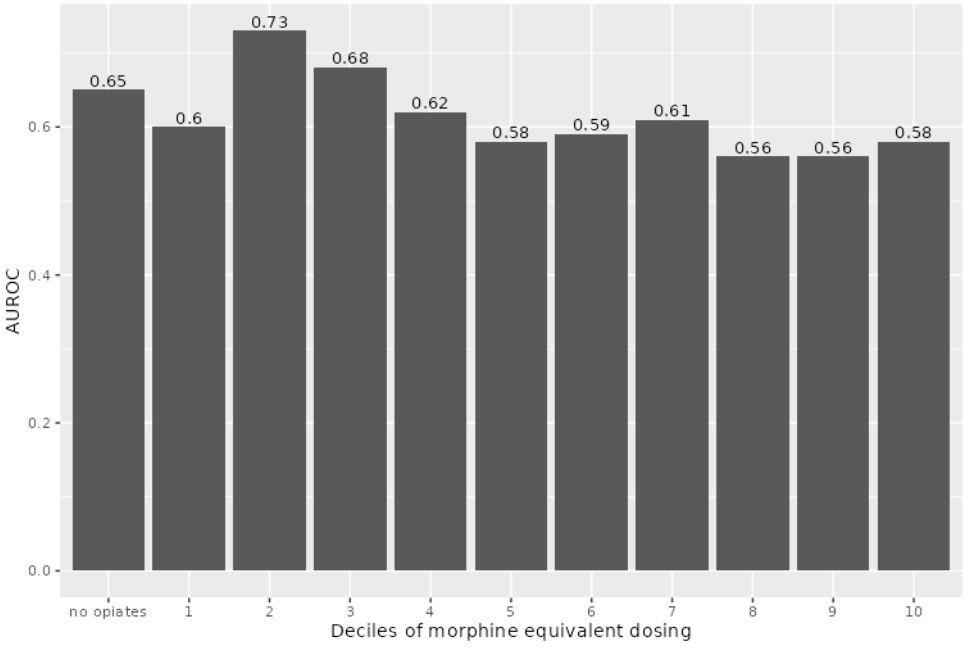


Supplementary Figure 10: AUROC for the PRE-DELIRIC model per decile of morphine equivalent dosing.


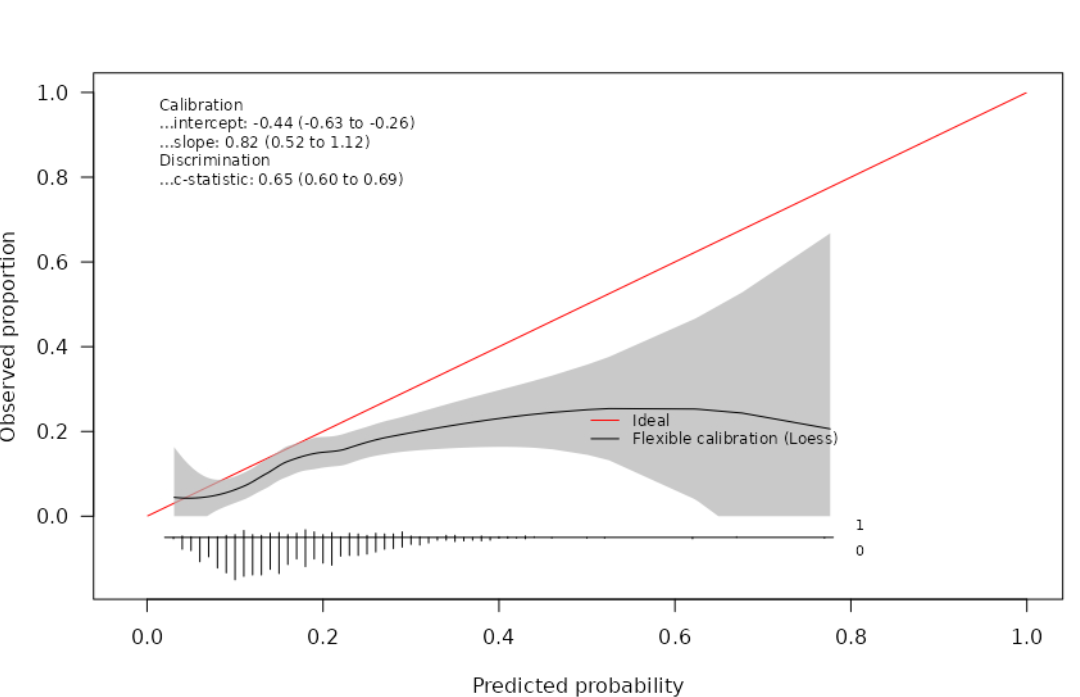


Supplementary Figure 11: Calibration plot for the PRE-DELIRIC model for the patients who do not receive opiates.


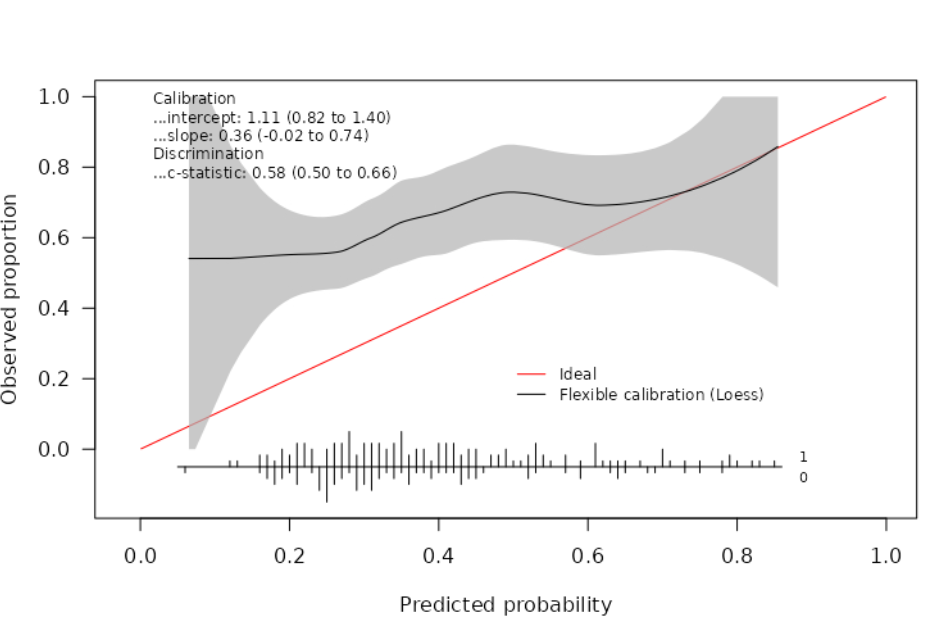


Supplementary Figure 12: Calibration plot for the PRE-DELIRIC model for the patients who are on the 10^th^ decile of morphine equivalent dosing (Supplementary Figure 7).

**References**

1. van den Boogaard, M., et al., *Development and validation of PRE-DELIRIC (PREdiction of DELIRium in ICu patients) delirium prediction model for intensive care patients: observational multicentre study.* BMJ, 2012. **344**: p. e420.
