# Supplementary material for: Validation of PREdiction of DELIRium in ICu patients (PRE-DELIRIC) model for ICU delirium in general ICU and patients with liver disease: a retrospective cohort study
Source: J Intensive Care. 2025 Jun 16;13:33. doi: 10.1186/s40560-025-00800-3 (PMC12168250; doi:10.1186/s40560-025-00800-3)
Supplement: Supplementary file 2 — Additional file 2. [file 40560_2025_800_MOESM2_ESM.docx]

Supplementary Table 1: PRE-DELIRIC predictors for ICU patients in Addenbrooke’s.

| **Predictor** |  |
| --- | --- |
| **Age mean in y (SD)** | 59 (17.1) |
| **APACHE-II score (SD)** | 16 (5.8) |
| **Coma category, n (%)** |  |
| 0. No coma: RASS-4/-5 maximum 8 hours | 2115 (63.9) |
| RASS-4/-5 for longer than 8 hours: |  |
| 1. With use of medication | 906 (27.3) |
| 2. Other (i.e. intra cerebral bleeding, post-resuscitation) | 9 (0.3) |
| 3. Combination (1+2) | 282 (8.5) |
| **Admission category, n (%)** |  |
| 1. Surgery | 1044 (31.5) |
| 2. Medical | 1901 (57.4) |
| 3. Trauma | 70 (2.1) |
| 4. Neuro | 297 (9.0) |
| **Infection, n (%)** | 2285 (68.9) |
| **Metabolic acidosis, n (%)** | 611 (18.5) |
| **Morphine equivalent category, n (%)** |  |
| 0. No morphine | 1120 (33.8) |
| 1. 0.01-7.1 mg | 378 (11.4) |
| 2. 7.2-18.6 mg | 69 (2.1) |
| 3. ≥18.7mg* | 1745 (52.7) |
| **Sedation, n (%)** | 1775 (53.6) |
| **Urea (mmol/L) median [IQR]** | 9.0 [6.0 - 14.2] |
| **Urgent admission, n (%)** | 2818 (85.1) |

*Although the van den Boogaard [1] PREDELIRIC paper had a cut-off of 200 mg, in CUH many patients had more than 200 mg of opiates, and thus they were included in this group.

Supplementary Table 2: Morphine category table using the restrictive “morphine use” definition that includes only morphine itself (as per van den Boogard [1]). The other predictors are as per Supplementary Table 1.

| **Predictor** |  |
| --- | --- |
| **Morphine equivalent category, n (%)** |  |
| 0. No morphine | 2830 (85.4) |
| 1. 0.01-7.1 mg | 411 (12.4) |
| 2. 7.2-18.6 mg | 46 (1.4) |
| 3. ≥18.7mg | 25 (0.8) |

Supplementary Table 3: PRE-DELIRIC model per morphine equivalent dosing decile.

| **Decile** | **AUROC (95% CI)** | **AUPRC** | **Sample size** | **Delirium incidence** |
| --- | --- | --- | --- | --- |
| No opiates | 0.65 (0.60-0.69) | 0.19 | 1,120 | 138 (12.3%) |
| 1 | 0.60 (0.50-0.70) | 0.24 | 220 | 36 (16.4%) |
| 2 | 0.73 (0.63-0.82) | 0.27 | 220 | 32 (14.5%) |
| 3 | 0.68 (0.61-0.76) | 0.45 | 219 | 73 (33.3%) |
| 4 | 0.62 (0.54-0.69) | 0.57 | 219 | 112 (51.1%) |
| 5 | 0.58 (0.51-0.66) | 0.51 | 219 | 102 (46.6%) |
| 6 | 0.59 (0.51-0.66) | 0.52 | 219 | 107 (48.9%) |
| 7 | 0.61 (0.53-0.69) | 0.58 | 219 | 114 (52.1%) |
| 8 | 0.56 (0.48-0.63) | 0.53 | 219 | 112 (51.1%) |
| 9 | 0.56 (0.48-0.64) | 0.59 | 219 | 126 (57.5%) |
| 10 | 0.58 (0.50-0.66) | 0.70 | 219 | 139 (63.5%) |

Supplementary Table 4: Comparison between morphine equivalent dosing in CUH and morphine dosing in the original PRE-DELIRIC paper (van den Boogaard et al. 2012 [1]).

|  | **Morphine Category** | | | |
| --- | --- | --- | --- | --- |
|  | 0  (No morphine) | 1  (0.01-7.1 mg) | 2  (7.2-18.6 mg) | 3  (18.7-200 mg) |
| Morphine equivalent dosing in CUH i.e. including the use of synthetic opioids. | 1,120 (33.8%) | 378 (11.4%) | 69 (2.1%) | 1,745 (52.7%) |
| Morphine use reported in the original study (van den Boogaard et al. 2012 [1]) | 884 (50.0%) | 75 (4.2%) | 558 (31.6%) | 251 (14.2%) |

**References**

1. van den Boogaard, M., et al., *Development and validation of PRE-DELIRIC (PREdiction of DELIRium in ICu patients) delirium prediction model for intensive care patients: observational multicentre study.* BMJ, 2012. **344**: p. e420.
